# ATRP-based synthesis and characterization of light-responsive coatings for transdermal delivery systems

**DOI:** 10.1088/1468-6996/16/3/034604

**Published:** 2015-05-08

**Authors:** Anja C Pauly, Katrin Schöller, Lukas Baumann, René M Rossi, Kathrin Dustmann, Ulrich Ziener, Damien de Courten, Martin Wolf, Luciano F Boesel, Lukas J Scherer

**Affiliations:** 1 Empa, Swiss Federal Laboratories for Materials Science and Technology, Laboratory for Protection and Physiology, Lerchenfeldstrasse 5, 9014 St. Gallen, Switzerland; 2 Ulm University, Institute of Organic Chemistry III, Macromolecular Chemistry and Organic Materials, Albert-Einstein-Allee 11, D-89081 Ulm, Germany; 3 Division of Neonatology, University Hospital Zürich, Frauenklinikstrasse 10, 8091 Zürich, Switzerland

**Keywords:** light-responsive materials, membranes, ARGET–ATRP, surface initiated polymerization, drug delivery

## Abstract

The grafting of poly(hydroxyethylmethacrylate) on polymeric porous membranes via atom transfer radical polymerization (ATRP) and subsequent modification with a photo-responsive spiropyran derivative is described. This method leads to photo-responsive membranes with desirable properties such as light-controlled permeability changes, exceptional photo-stability and repeatability of the photo-responsive switching. Conventional track etched polyester membranes were first treated with plasma polymer coating introducing anchoring groups, which allowed the attachment of ATRP-initiator molecules on the membrane surface. Surface initiated ARGET–ATRP of hydroxyethylmethacrylate (where ARGET stands for activator regenerated by electron transfer) leads to a membrane covered with a polymer layer, whereas the controlled polymerization procedure allows good control over the thickness of the polymer layer in respect to the polymerization conditions. Therefore, the final permeability of the membranes could be tailored by choice of pore diameter of the initial membranes, applied monomer concentration or polymerization time. Moreover a remarkable switch in permeability (more than 1000%) upon irradiation with UV-light could be achieved. These properties enable possible applications in the field of transdermal drug delivery, filtration, or sensing.

## 1. Introduction

Stimuli-responsive materials have found widespread applications as the need to create novel smart systems often requires materials, which can change their chemical and/or physical properties by changing an external parameter [1]. A change of temperature, pH, oxidation state, humidity or light has been shown to be capable of significantly changing the properties of materials [2].

In the research area of artificial membranes the control over mass transfer rates is one of the most important challenges [3]. Stimuli-responsive membranes, which change their permeability upon an external stimulus, are promising candidates. These membranes find potential applications in the field of drug delivery systems [4]. For example, a drug delivery system for preterm neonates on the basis of a stimuli-responsive membrane has the potential to overcome problems with peaks in drug concentration by oral or venous medication [5]. In addition the administering of drugs by transdermal delivery systems reduces the stress for the very sensitive neonates. Although different stimuli have been reported to switch the permeability of membranes [6, 7], for applications close to the human body, light is the ideal stimuli. Light can be applied rapidly, remotely and reversibly and additionally, light is a clean stimulus that can be easily focused on small and defined areas [8]. Spiropyran (SP) is one of the most widely used photo-responsive organic molecules. SP undergoes a structural isomerization upon irradiation with UV-light; this structural isomerization is accompanied with an increased polarity and a change in color of the SP compound. This photo-isomerization is completely reversible by irradiation with white light [9, 10]. Additionally, no cell toxicity of SP in solution at concentrations below 10^−6^ M are observed [11], which makes SP a suitable candidate for the synthesis of photo-responsive membranes in transdermal drug delivery systems. Under UV-irradiation the colorless acyclic SP undergoes a reversible heterocyclic ring-opening reaction leading to the more polar and colored merocyanine state [10, 12]. Visible light triggers the ring closing reaction back into the initial SP state. Only few examples of photo-responsive membranes based on SP have been published [12–14].

Preterm neonates are susceptible to apnea since their respiratory system is not fully developed yet. Therefore caffeine was applied to the neonates to treat and prevent apnea [15]. Also the skin (*Stratum corneum*) of preterm neonates is not fully developed and therefore possesses only a minimal barrier for the delivery of caffeine [16]. The caffeine concentration in the body is not only dependent on the permeability of the membrane, but is also influenced by the resistance of the skin towards caffeine. Ideally the membrane should have a responsive ‘on–off’ behavior, where the permeability of the membrane in the off-state is much below the resistance of the skin whereas in the on-state the permeability is far in excess of the permeability of the skin. To adjust the permeability of the membrane to the permeability of the skin, a method to obtain photo-responsive membranes with tunable permeability is highly desired.

We have already reported on the use of photo-responsive membranes or coatings in smart caffeine release systems [13, 17–19]. Therefore commercial track-etched membranes were modified with plasma-induced surface-initiated free radical polymerization of 2-hydroxyethylmethacrylate (HEMA) and SP as photo-responsive groups. A significant increase in the caffeine permeability was observed when changing from white LED light to UV-light irradiation. However, the overall permeability could only be modified by changing the initial membrane thickness and the pore diameter of the membranes. An exact tuning of the permeability rate was difficult to perform due to the limited available types of membranes. Another drawback of this approach is the lack of control over the polymerization process, therefore the thickness of the grafted polymer layer cannot be predicted and polymer chains with a large polydispersity index were obtained. Living polymerization methods offer more control over the polymer layer and the size of the grafted polymers can therefore be easily adjusted. One such method, suitable for the grafting of (meth)acrylates on surfaces, is atom transfer radical polymerization (ATRP) [20]. In ATRP the propagating polymer chain ends are reversibly deactivated to form dormant species. Due to the reduction of the radical concentration, termination reactions are suppressed enabling a living polymerization process. The dormant species are intermittently reactivated in a catalytic manner [21].

Grafting of poly(oligo (ethylene glycol) methacrylates) on the surface of track etched membranes by surface initiated ATRP has recently been shown to create thermo-responsive membranes [22]. At low temperatures the polymers are swollen and the pores are blocked; when the temperature is increased, the polymer chains collapse resulting in more opened pores. However, achieving a steric hindrance can be demanding if the ratio of pore diameter to brush thickness is large. Thus, a thick coating is needed for the successful gating of a membrane by steric interaction. On the other hand, the layer should not be too large either, since otherwise no permeability change would be observed [22]. An alternative approach which has been reported in the literature is to change the hydrophilicity of a membrane by an external trigger; this effect leads to a reversed permeability change [17–19]. The permeability of the membrane is influenced by the wetting behavior of the pores. If the pore surface is hydrophilic, aqueous solutions pass through easily. If the pore surface is changed to a hydrophobic state, less aqueous solution is able to pass through [23]. SP fulfills the requirement of the hydrophobic-hydrophilic transition and thin coatings are already sufficient to influence the surface tension of a membrane.

In this paper we expand our previous work on photo-responsive coatings by using a controlled radical polymerization technique. We developed membranes with a switchable permeability by grafting poly(hydroxyethylmethacrylate) (pHEMA) on the surface of track-etched membranes via surface initiated ATRP. Due to the controlled polymerization conditions of the pHEMA layer on the membrane surface, the permeability of the membranes could be tuned by the initial pore diameter of the membrane, applied monomer concentration and polymerization time, allowing a control over the permeability properties by adjusting the membrane morphology.

## 2. Experimental procedures

### 2.1. Materials

All reagents were purchased from Sigma-Aldrich, Acros or Fluka and used as received unless stated differently. Polyester membrane filters (PETE, 0.1 or 0.2 *μ*m pore diameter, 25 mm diameter, Sterlitech) were washed with dichloromethane for 3 h prior to use. Methyl-*tert*-butylether (MTBE) was dried over molecular sieves (3 Å), *α*-bromoisobutyrylbromide was distilled under reduced pressure, 2-hydroxyethylmethacrylate (HEMA) was purified as reported in literature [24]. HEMA was dissolved in deionized water and washed three times with hexane. The water phase was saturated with sodium chloride (NaCl) and extracted three times with diethylether (Et_2_O). The combined organic phases were dried with magnesium sulfate (MgSO_4_). After removing of the solvent HEMA was distilled under reduced pressure.

### 2.2. Characterization

The ^1^H and ^13^C nuclear magnetic resonance (NMR) spectra were recorded using a Bruker Avance 400 NMR instrument. The chemical shifts relative to the remaining resonances of the corresponding solvent are given in parts per million (ppm). IR spectra were acquired with a Bio-Rad FTS-6000 Fourier transorm infrared (FTIR) spectrometer equipped with an attenuated total reflectance unit (Golden Gate). Scanning electron microscopy (SEM) measurements were performed using the Hitachi S-4800 FE-SEM (field emission SEM), using an acceleration voltage of between 2 and 10 kV. They were carried out at ultra-high vacuum. Before the measurement the sample was coated with thin (5 nm) gold/palladium layer in a sputter coater (Leica ACE 600). X-ray photoelectron spectroscopy (XPS) data were acquired using a PHI LS 5600 machine equipped with a standard MgK*α* x-ray source. The energy resolution of the spectrometer was set to 0.8 eV/step at pass energy of 187.85 eV for survey scans and 0.25 eV/step and 58.7 eV pass energy for more detailed scans. The x-ray beam was operated at a current of 25 mA and an acceleration voltage of 13 kV. Charge effects were corrected using the carbon C1s energy of 284.5 eV. Ellipsometry measurements were performed with an ellipsometer M-2000F EC400. The relative humidity was measured with EL-USB-2 sensor from Lascar Measurement description. Inductively coupled plasma optical emission spectrometry (ICP–OES) measurements were performed on a Perkin Elmer, optima 3000. Solid-state UV-measurements were performed on a Lambda 19 spectrometer from Perkin Elmer in reflection or transmission mode. UV spectra in solution were recorded on a Varian 50Bio/50MPR. The UV irradiation was carried out with a UV-light emitting diode (intraLED 3 from Volpi, 386 nm, 1.5 mW cm^−2^), the white LED light source was an intra LED 3 Volpi (400–700 nm, 500 lumens). Permeability measurements were performed in a Franz diffusion cell from SES Analysesysteme with a receptor volume of 12 mL and an orifice area of 1.77 cm^2^.

### 2.3. Synthesis of *3*-(*3*',*3*'-dimethyl-*6*-nitrospiro[chromene-*2*,*2*'-indolin]-*1*'-yl)propanoic acid (SP-COOH)

3-(3',3'-dimethyl-6-nitrospiro[chromene-2,2'-indolin]-1'-yl)propanoic acid (SP-COOH) was synthesized according to a previous published procedure [17]. The two-step reaction is sketched in scheme S1.

#### 2.3.1. Synthesis of *1*-(*3*-carboxyethyl)-*3*,*3*-dimethyl-*2*-methyleneindoline

Indoline (1.0 eq.) was dissolved in methyl-ethylketone (MEK) and 3-iodopropanoic acid (1.1 eq.) was added. The reaction was stirred under reflux for 12 h under inert gas atmosphere. After cooling to room temperature the precipitate was isolated by vacuum filtration and washed with cold hexane followed by washing with Et_2_O. The resulting 1-(3-carboxyethyl)-3,3-dimethyl-2-methyleneindoline was obtained as a light brown solid in 99% yield. NMR data were as follows: ^1^H NMR (400 MHz, DMSO) *δ* (ppm) = 8.05–7.90 (*m*, 1H), 7.83 (*dd*, *J* = 4.0, 2.1 Hz, 1H), 7.61 (*dd*, *J* = 5.8, 3.1 Hz, 1H), 4.64 (*t*, *J* = 7.0 Hz, 1H), 2.97 (*t*, *J* = 7.0 Hz, 1H), 2.86 (*s*, 1H), 1.52 (*s*, 1H). ^13^C NMR (101 MHz, DMSO) *δ* 197.88, 171.50, 141.73, 140.80, 129.32, 128.89, 123.47, 115.55, 54.24, 43.55, 31.09, 21.86, 21.74, 14.44. Here DMSO stands for dimethyl sulfoxide, s for singlet, t for triplet and dd for doublet of doublets.

#### 2.3.2. Synthesis of *3*-(*3*',*3*'-dimethyl-*6*-nitrospiro[chromene-*2*,*2*'-indolin]-*1*'-yl)propanoic acid (SP-COOH)

All glassware were pre-dried and wrapped with aluminum foil. To 1-(3-carboxyethyl)-3,3-dimethyl-2-methyleneindoline (1.0 eq.) in MEK piperidine (1.1 eq.) and benzaldehyde (1.0 eq.) were added in one portion. The reaction mixture was stirred for 3 h at 80 °C. After cooling to room temperature the flask was stored for 12 h without stirring and cooled to 0 °C. The precipitated material was isolated by vacuum filtration and washed with cold MEK and methanol (MeOH). The resulting SP-COOH was obtained as a yellow powder in 63% yield. NMR analysis: ^1^H NMR (400 MHz, DMSO) *δ* (ppm) = 12.22 (*s*, 1H), 8.20 (*s*, 1H), 7.99 (*d*, *J* = 11.7 Hz, 1H), 7.20 (*d*, *J* = 10.4 Hz, 1H), 7.10 (*d*, *J* = 7.3 Hz, 2H), 6.85 (*d*, *J* = 9.0 Hz, 1H), 6.79 (*t*, *J* = 7.4 Hz, 1H), 6.65 (*d*, *J* = 8.0 Hz, 1H), 5.98 (*d*, *J* = 10.4 Hz, 1H), 3.60–3.21 (*m*, 3H), 2.66—2.26 (*m*, 4H), 1.17 (*s*, 7H), 1.06 (*s*, 3H).

^13^C NMR (101 MHz, DMSO) *δ* (ppm) = 172.85, 159.07, 146.10, 140.54, 135.64, 128.15, 127.63, 125.70, 122.79, 121.77, 119.22, 118.83, 115.48, 106.63, 106.49, 52.41, 38.85, 33.15, 25.57, 19.45.

### 2.4. Plasma polymer coating of the membranes

The raw membranes were initially dipped in dichloromethane for 3 h at room temperature. This treatment removes possible coatings or impurities that remain from the manufacturing process.

An amine group containing plasma polymer coating was prepared by a plasma polymerization process: 270 sccm ethylene, 250 sccm ammonium and 25 sccm argon were activated by 635 W at a pressure of 0.1 mbar in a batch reactor [25]. Both sides of the membranes were treated, each for 30 min. The membranes were stored in a desiccator over molecular sieves.

### 2.5. *α*-Bromoisobutyryl bromide binding on the membranes

To a solution of 11 mL MTBE and 0.25 mL thriethylamine (NEt_3_) a membrane treated with a plasma polymer coating was added under inert gas atmosphere at 0 °C. Then 0.325 mL *α*-bromoisobutyryl bromide was added drop wise to the solution via syringe. The closed flask was transferred to a lab shaker and the mixture was shaken for 3 h at 100 rpm at room temperature.

Afterwards the membranes were washed consecutively with MTBE, MeOH and water. An ultrasonic bath was used for each step for 5 min. Hereafter the membranes were dried in a desiccator over molecular sieves overnight. FTIR analysis: *ν* (cm^−1^) = 2965, 2914, 2860 (aliphatic C–H), 1707 (C=O), 1236 (C–O).

### 2.6. Surface initiated ARGET–ATRP of HEMA on the membranes (where ARGET stands for activator regenerated by electron transfer)

A stock solution of copper (II) bromide (CuBr_2_, *c* = 1.74 mmol L^−1^) in water/methanol (1/1 vol%) was degassed by bubbling argon through the solution under stirring for 1 h. In another flask a solution with a total volume of 10 mL with 2,2′-bipyridine (bipy, 8.96 mmol L^−1^), ascorbic acid (AscA, 28.39 mmol L^−1^) and a variable amount of HEMA (0.2–25 vol%) in water/methanol (1/1 vol%) was degassed by bubbling argon through the solution under stirring for 1 h. The membrane functionalized with α-bromoisobutyryl bromide was added. An additional flask with a solution consisting of HEMA in the applied concentration in water/methanol (1/1 vol%) was prepared. It serves as reference for the solution’s temperature and is equipped with a temperature sensor. The flasks were shaken at a speed of 100 rpm and heated to a temperature of 60 °C. After reaching 60 °C 1 mL of copper bromide stock solution was added and the flasks were shaken for 20 h at 60 °C. After the membranes were removed and washed with methanol and water in an ultrasonic bath for 5 min, they were dried in a desiccator over molecular sieves overnight. FTIR analysis: *ν* (cm^−1^) = 3373 (OH), 2955, 2918, 2851 (aliphatic C–H), 1715 (C=O), 1236 (C–O), 1163 (C–O).

#### 2.6.1. Ellipsometry measurements of the membranes

For ellipsometry measurements silica wafers were prepared. The wafers were treated analog to the membranes (plasma polymer coating, initiator binding and surface initiated polymerization of HEMA). All ellipsometry measurements were made by SuSoS AG (Dübendorf) and the descriptions of the setup and method of measurement was provided by SuSoS. The thickness of the polymer layer synthesized on a silicon wafer in dry and wet air is determined. Therefore the thickness of the polymer layers were determined as a function of the humidity of the air. The samples were exposed to the lowest (10%) respectively highest (90%) possible humidity values. The samples were placed in the measurement chamber that was flooded with dry nitrogen. After 5 min the ‘dry’ thickness was determined. Then the samples were exposed to wet nitrogen and after 5 min the wet thickness was measured.

#### 2.6.2. ICP–OES measurement for determining the copper concentration in the membranes

To determine the copper content in the membranes ICP–OES measurements were performed. Therefore the membrane was dissolved in 3.5 mL HNO_3_ and transferred to the inner container of the disintegration device. 1 ml H_2_O_2_ was added to the outer container so that it can evaporate to the inner container during the disintegration, which takes place in a microwave oven (Milestone, mls 1200 mega). The energy of the microwave was controlled by the program. In this case the program was: 1 min 500 W, 1 min 0 W, 2 min 500 W, 1 min 0 W, 3 min 500 W, 1 min 0 W, 4 min 500 W. The resulting solution was transferred to a 50 mL flask and filled up to 50 mL with water. This solution was detected with the ICP–OES. For this measurement 10 membranes were used to have a basis amount of substance. The determined copper content was 42 ppm. This detected amount of copper is in a comparable range as found in literature [26]. If the resulting amount of copper is calculated per membrane, the incorporated amount is 259 ng/ membrane (2.35×10^−4^ wt%).

#### 2.6.3. Polymerization kinetics

For a concentration of 10 vol% HEMA and a membrane with a pore size of 0.2 *μ*m the kinetic of the surface initiated ARGET–ATRP of HEMA was explored. For that membranes were polymerized in one flask and removed after different time intervals (15, 30, 60, 120 and 240 min).

### 2.7. Post-polymerization modification with SP

In a round bottom flask 100 mg SP-COOH was dissolved in 12 mL N,N′-dimethylformamide (DMF, water free) with an ultrasonic bath. 55 mg dicyclohexylcarbodiimide (DCC) and 33 mg 4-dimethylaminopyridine were added under dry conditions. After adding the membrane the flask was shaken with 100 rpm at room temperature overnight. The membranes were washed with methanol and water and dried in the desiccator over molecular sieves.

#### 2.7.1. Determination of the SP content

The coated membrane was dunked into 2 mL 1 M NaOH solution. It was stirred until the membrane was completely dissolved. The absorption of the resulting solution containing 2-hydroxy-5-nitrobenzaldehyde (decomposition product of SP) was measured at 389 nm (*V* = 200 *μ*L). It was assumed that SP is quantitatively decomposed. The SP content in the membranes were calculated with the following calibration curve:

*c*_SP_ (mMol L^−1^) = 0.11377 *x* + 0.00448 (*x*: measured absorption).

#### 2.7.2. Ring closing kinetics

The solid state UV/Vis measurement of the membranes was carried out in reflection mode. The baseline (*R* = 100%) was measured without a membrane. Then spectra of the membrane after illumination with white LED light for 3 minutes (100%), illumination with UV-light (50%) for 1 minute with a distance of 5 cm were recorded. The membrane remained in the instrument for the whole measurement. To find out about the ring-closing kinetic of the SP under dark conditions, 30 spectra were recorded during 210 min.

#### 2.7.3. Reversibility of color switching

The solid state UV/Vis measurement of the membranes was carried out in transmission mode. The absorption maximum of the excited membrane was found to be at 560 nm. Therefore the area between 561–559 nm was scanned in 0.2 nm steps. Out of the 11 points the average was calculated using the arithmetic mean. For the unexcited state the membrane was illuminated with the white LED light source (100%, 2 min) the excitation was carried out with the UV-light source (50%, 1 min). The membrane remained in the UV-Vis for the whole measurement. The switch was performed 40 times.

#### 2.7.4. Fading rate

The solid state UV/Vis measurement of the membranes was carried out in reflection mode. First the spectra of the membranes irradiated with white LED light were measured, then the membranes were irradiated with UV-light (50%) permanently. Experiments typically lasted 350 min, during which the membranes were scanned 30 times. During the measurement the illumination was stopped. After the measurement the illumination and the time measurement were continued.

### 2.8. Permeability of the membranes

The permeability of the membranes were measured with a Franz diffusion cell. The receptor volume is 12.0 mL and the orifice area was 1.77 cm^2^. Before placing the membranes in the Franz cell, they were either irradiated with UV-light or white LED light for 1 min. Mass transfer rates of caffeine were measured under UV irradiation and at room light. After filling the receptor chamber with water (12.0 mL), the membrane was fixed in the diffusion cell. The donor chamber was charged with a caffeine solution (93 mM; 3.0 mL). Samples (200 *μ*L) were collected from the receptor part of the cell regularly 14 times during 480 min of the experiment. The caffeine concentrations of these samples were assigned by measuring its UV absorption at 293 nm with the following calibration curve: *c*_caff_ (mM) = (*y*−0.0468) (0.7048)^−1^ (*y*: measured absorption). The permeability of a membrane at a given caffeine concentration is proportional to the molecular flux *F* of aqueous caffeine through the membrane, calculated according to 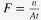 where *n* represents the amount of material, *A* the orifice area and *t* the permeation time. All membranes were stored in water to precondition the membranes for the permeability measurements.

## 3. Results and discussion

### 3.1. Membrane modification

**Scheme 1.**
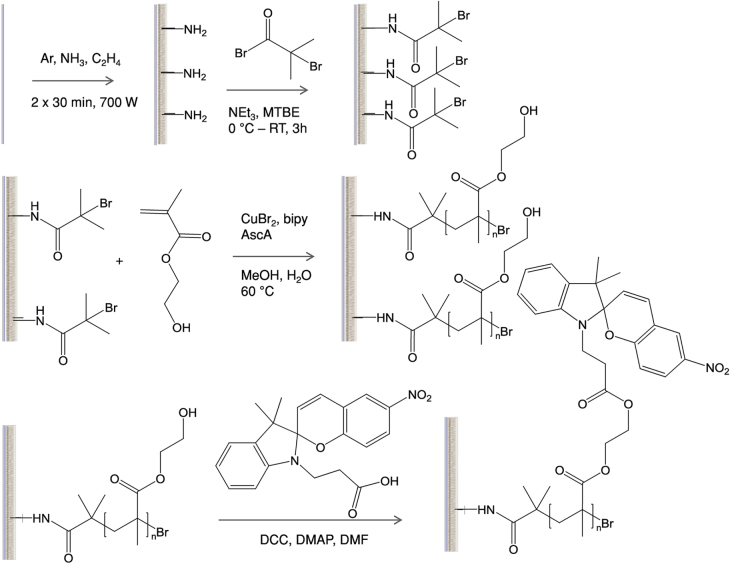
Overview of the reaction steps from the raw membrane to plasma polymer coating (top left), ATRP-initiator binding (top right), surface-initiated polymerization of HEMA (middle) and functionalization with spiropyran (SP, bottom).

Track etched polyester membranes with different pore diameters (0.2, 1 and 2 *μ*m) were modified according to scheme 1. For the sake of clarity the modification of only one side of the membrane is shown, however, both sides of the membranes were modified. First the surface of the membranes were activated by introducing amine groups, these amine groups were used to attach an ATRP initiator, then surface initiated ATRP of 2-hydroxyethylmethacrylate (HEMA) was performed and finally the membranes were functionalized with SP in a post-polymerization modification step.

#### 3.1.1. Activation of membrane surface

The track etched polyester membranes were commercially available. They were initially washed with dichloromethane to remove possible surfactants or coatings remaining from the production process. The SEM pictures of the membrane before and after washing are shown in the supporting information (figure S1), they show a clear change in membrane morphology before and after the washing process; specifically, the pore size decreases after washing with dichloromethane. The membranes after the washing process possess a plain regular surface with a well-defined pore diameter with dimensions in agreement with the pore diameter stated by the manufacturer.

To enable a surface-initiated ATRP on the membrane surface, plasma polymer coating introducing primary amine groups as anchor groups on the membrane surface was used. Plasma polymer coating is known to produce smooth thin and adherent polymer films [27]. The applied gas mixture of ethylene and ammonia is already known to yield thin films with a comparably high concentration of primary amine groups [28]. Subsequent reaction of these amine groups with α-bromoisobutyryl bromide in solution yielded a membrane where a tertiary bromine group as ATRP initiator is attached to the surface.

The modification of the membrane was followed by XPS measurements, the results being shown in table 1. After the plasma polymer coating (lane in the table named as NH_2_) a relative atomic nitrogen concentration of 11.7% could be observed (signal of nitrogen 1s orbital), which indicates a successful functionalization of the membranes with amine groups. After the coupling of the ATRP initiator molecule the amount of nitrogen was slightly lowered, whereas the oxygen content was markedly increased revealing a possible oxidation of the amine groups of the plasma polymer coating [28] under the reaction conditions of the initiator binding. Nevertheless, enough amine groups were still present for the successful initiator binding; the presence of a relative atomic bromine content of 1.6% (signal of bromine 3p orbital) was clearly detected in XPS measurements confirming the presence of *α*-bromoisobutyryl groups on the membrane surface. Upon deposition of the plasma polymer layer small changes in the membrane morphology and a reduction in the pore diameter was observed by SEM. No further change in the pore diameter or membrane morphology was detected after initiator binding (figure S1).

**Table 1.** XPS measurements of the membranes (0.2 *μ*m) before any treatment, after plasma polymer coating, coating with the initiator layer and with the pHEMA layer.

Membrane	C 1s (%)	O 1s (%)	N 1s (%)	Br 3p (%)
Raw	75.7	24.3	0	0
NH_2_	84.4	3.9	11.7	0
Initiator	74.0	13.7	10.7	1.6
HEMA (1%)	74.3	24.3	1.4	0
HEMA (25%)	66.6	33.4	0	0
HEMA(cal.)	66.7	33.3	0	0

#### 3.1.2. Surface initiated polymerization of HEMA on the membrane

The polymer layer on the membrane was synthesized via surface initiated ARGET–ATRP. This living radical polymerization technique offers a good control over the polymerization process and over the grafted polymer layer. In conventional ATRP high catalytic concentrations of Cu(I) are needed for a living polymerization as the Cu(I) is oxidized towards Cu(II) as a side effect of radical chain termination processes. In the ARGET–ATRP the copper catalyst is added in the stable oxidized Cu(II) state and reduced to the active Cu(I) species *in situ* by a reducing agent. The presence of a reducing agent in ARGET–ATRP maintain an appropiate Cu(I)/Cu(II) balance enabling a living polymerization with a considerably lowerd copper concentration in the ppm range [29]. Here as reducing agent ascorbic acid is used in great excess, in this way the polymerization becomes less sensitive toward oxygen as Cu(I) can be reproduced during the polymerization. As monomer HEMA was used, as shown in scheme 1. This monomer was chosen because ARGET–ATRP of HEMA was already reported to yield well defined polymers with a controllable molecular weight [30]. Moreover, the polymer pHEMA is known to be biocompatible [6, 31] and therefore is either in use or has been proposed as a material for contact lenses [32] or bone cements [33]. In addition pHEMA enables further modification steps due to the hydroxyl group in the polymer side chain. The FTIR spectra of the membrane before and after polymerization of HEMA are shown in figure S2. In both spectra characteristic peaks around 2900 cm^−1^ corresponding to aliphatic C–H bonds and at 1715 cm^−1^ corresponding to the C=O carbonyl bond can be seen. In the FTIR spectra of the membrane after polymerization a new broad peak around 3400 cm^−1^ appears, which is related to the hydroxyl groups of pHEMA, which proofs a successful grafting of pHEMA on the membrane surface.

**Figure 1.**
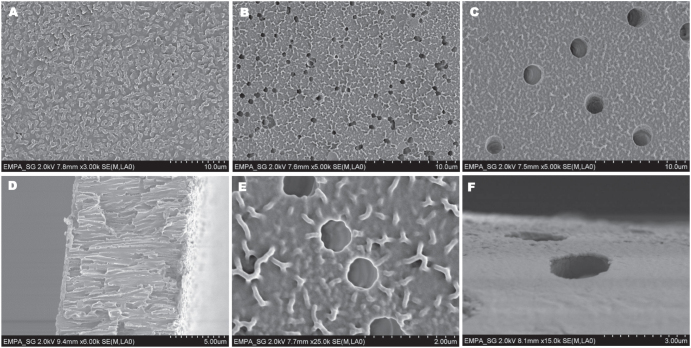
SEM images of membranes 0.2 *μ*m, *c*_HEMA_ 10% (A), 1 *μ*m, *c*_HEMA_ 10% (B), 2 *μ*m, *c*_HEMA_ 10% (C), cross-section 0.2 *μ*m, *c*_HEMA_ 10% (D), 1 *μ*m, *c*_HEMA_ 10% (E) and 2 *μ*m, *c*_HEMA_ 10% (F).

As mentioned above, one drawback of ATRP is the amount of copper remaining in the polymer, what limits the application of these polymers due to the toxicity of copper [34]. The amount of copper is reduced by the use of ARGET–ATRP. The remaining copper content in the membranes was measured by ICP–OES and is amounted to 260 ng copper per membrane (0.0002 wt%). However, this small amount has no negative effect on the human body limiting possible biomedical applications. Actually, the human body requires the presence of a small concentration of copper [34]. Three different membranes (with pore diameter 0.2, 1 and 2 *μ*m) were coated with a pHEMA layer via a surface initiated ARGET–ATRP, the SEM pictures of the three different modified membranes are shown in figures 1(A)–(C). All modified membranes showed a clear change in morphology after polymerization, indicating a successful covering of the membrane surface with a pHEMA layer. For the membrane with a pore diameter of 0.2 *μ*m most pores were completely covered by a homogenous polymer layer. In the case of membranes with a pore diameter of 1 and 2 *μ*m, the porous structure of the membranes was still present after polymerization. It can be seen in the cross-section SEM pictures of the membranes (figures 1(D) and S3) that the porous structure is still present and only the surface of the membrane is covered with the polymer layer. The figures 1(E) and (F) show SEM pictures of the pHEMA layer on the membranes with a pore diameter of 1 and 2 *μ*m in a higher magnification, clearly displaying the pHEMA layer on the membrane surface.

Next to the variable pore diameter of the membranes also the influence of the monomer concentration during the ARGET–ATRP on the formed polymer layer thickness was examined. HEMA concentrations of 0.2, 1, 10 and 25% were tested. In a living polymerization the degree of polymerization equivalent with the molecular weight increases with increasing monomer concentration under identical polymerization conditions [35]. In addition, in a surface initiated polymerization the thickness of the polymer layer increases with increasing degree of polymerization under constant grafting densities [36]. Therefore, for the first instance the increase in weight of the membranes after polymerization was used to evaluate the layer thickness of pHEMA on the membrane. The results are presented in table 2. In general, the weight increase of the membranes due to the formation of grafted polymer was proportional to the used HEMA concentration. The pHEMA layer on membranes with a pore diameter of 0.2 *μ*m increases from 0.38 wt% with an applied HEMA concentration of 0.2%–48 wt% with a HEMA concentration of 25%. By comparing the amount of pHEMA layer on the membranes with a variable pore diameter, it turned out that the amount of pHEMA layer decreases with increasing pore diameter of the membranes. The highest observed amount of pHEMA layer of 48 wt% was obtained with the membranes with a pore diameter of 0.2 *μ*m; in comparison only 7 wt% weight increases after grafting of pHEMA was obtained with the membranes with 2 *μ*m pore diameter with the same applied HEMA concentration of 25%. Due to the anisotropy of the plasma process no amine groups are formed inside the pores of the membrane [25] and the pHEMA layer is only formed on the membrane surface and not in the pores. Therefore the surface accessible for the grafting process of pHEMA on the membranes with a bigger pore diameter is decreased compared to a smaller pore diameter resulting in a lower weight increase of the membranes with a pore diameter of 2 *μ*m after polymerization.

**Table 2.** Overview of the modified membranes and the pHEMA and spiropyran content.

Membrane	*c*_HEMA_ (vol%)	Polymerization time (min)	Poly(HEMA) content (%)^a^	SP content (%)^b^	*F*_RL_^c^	*F*_UV_^c^	Δ*F* (%)^d^
0.2 *μ*m	0	0	0	0	1.6	-^e^	–
0.2 *μ*m	0.2	1200	0.38	-^e^	6.2	9.3	50
0.2 *μ*m	1	1200	3.2	-^e^	8.9	10	13
0.2 *μ*m	10	15	12	-^e^	0.96	1.2	30
0.2 *μ*m	10	60	19	-^e^	0^f^	1.3	>10^3^
0.2 *μ*m	10	120	25	3.2	-^e^	-^e^	–
0.2 *μ*m	10	240	28	-^e^	0^f^	0.11	>10^3^
0.2 *μ*m	10	1200	31	-^e^	0.031	0.19	530
0.2 *μ*m	25	1200	48	3.5	0.41	0.49	20
1 *μ*m	0	0	0	0	40	-^e^	–
1 *μ*m	10	1200	17	5.7	4.8	6.1	27
1 *μ*m	25	1200	31	8.8	0.21	0.34	62
2 *μ*m	0	0	0	0	39	-^e^	–
2 *μ*m	10	1200	4.9	3.9	9.0	14	56
2 *μ*m	25	1200	7.2	3.5	1.4	1.6	14

a Determined by measuring the average membrane mass before and after polymerization.

b Determined by decomposing of the spiropyran in the membrane and measuring the concentration of 2-hydroxy-5-nitrobenzaldehyde (decomposition product).

c Units: 10^−9^ mol s^−1^ cm^−2^.

d *ΔF* = 100 (*F*_UV_ − *F*_RL_)/*F*_RL_.

e Was not determined.

f Permeability below detection limit.

**Figure 2.**
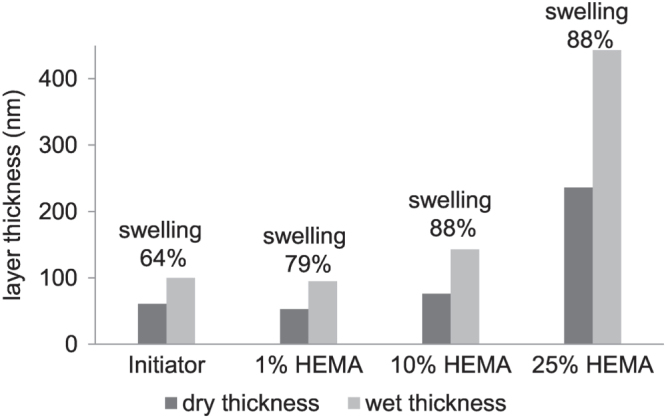
Results of ellipsometry measurements of the initiator and the pHEMA layer on silicon wafer (*c*_HEMA_ = 1%, 10% and 25%).

In addition, the grafting of pHEMA on the membranes was followed by XPS. The spectra of the membranes coated with 1% and 25% of HEMA are shown in table 1. After polymerization a change in the surface composition was detected. The peaks related to bromine are not detected anymore. Bromine should be present at the chain end after ATRP, but the polymerization was performed in aqueous solution, where the terminal bromine atom at the polymer chain end is easily hydrolyzed [29]. When a solution with only 1% HEMA is polymerized a comparably thin (<10 nm) pHEMA layer is formed; therefore the signals related to the membrane surface were still present in the XPS spectra, whereas the signals of the membrane grafted with 25% HEMA were in accordance with the theoretical value of pHEMA, indicating a thicker polymer layer. These findings are in agreement with ellipsometry measurements performed on silicon wafers. The silicon wafers were treated analog to the membranes: initially plasma polymer coating, then initiator binding and finally grafting of HEMA was performed. The use of silicon wafers as substrate for the characterization of surface grafted polymers is a widely accepted method in literature [37]. Also the comparison with thin porous membranes was recently described [38]. The thicknesses of the membranes after initiator binding and after polymerization with variable HEMA concentrations (1, 10 and 25%) are displayed in figure 2. The ellipsometry results confirm that there is a correlation between the applied HEMA concentration and the thickness of the polymer layer. The thickness of the initiator layer was in the same range of the polymer layer with 1% of HEMA (around 60 nm), whereas a clear increase in polymer layer thickness could be detected after polymerization with 10% HEMA (76 nm) or 25% HEMA (236 nm).

In addition the ability of the polymer layer to swell under humid conditions was tested by ellipsometry. Therefore the water uptake of the membranes was measured. The membrane with plasma polymer coating and attached ATRP-initiator already possess the ability to swell under humid conditions. The water uptake was 64%; which further increased with increasing polymer layer thickness up to a threshold of 88% for HEMA concentrations of 10 and 25%. It is very likely, that also the pHEMA layer grafted from the membrane swells under humid conditions which is supposed to have an effect on the permeability of the resulting membrane as discussed later.

**Figure 3.**
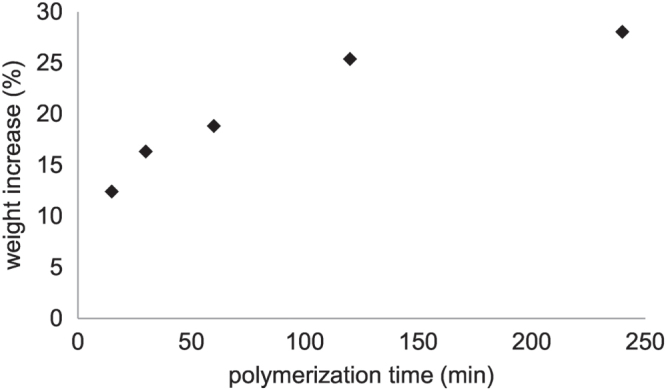
Polymerization kinetics (*c*_HEMA_ = 10%) on a membrane (0.2 *μ*m).

To explore the surface initiated ARGET–ATRP of HEMA on porous polyester membranes in more detail the weight increase of the membranes in dependence of the polymerization time was determined and shown in figure 3. For a polymerization time below 2 h a linear increase in weight was observed, enabling a control over the amount of coated polymer on the membrane surface. After four hours no marked increase in membrane weight could be detected, indicating the presence of chain termination reactions in the polymerization process. After four hours a weight increase of 28 wt% and after 20 h of 31 wt% was achieved. The chain termination reaction are mainly caused by the successive loss of active chain ends due to hydrolysis of the bromine end groups as already indicated by XPS measurements. In summary, the surface initiated ARGET–ATRP led to a grafted pHEMA layer on the membrane surface, where the polymer layer thickness could be controlled either by the applied HEMA concentration or by the polymerization time.

#### 3.1.3. Post-polymerization modification of the membrane with SP

**Figure 4.**
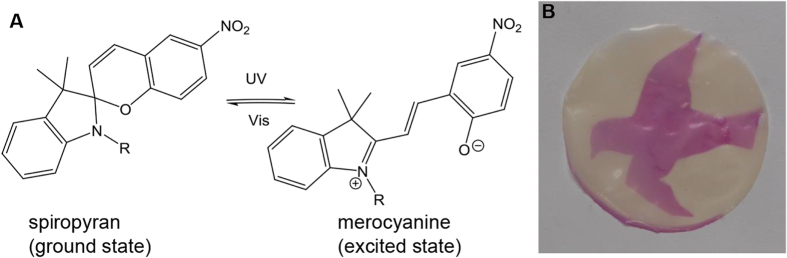
Spiropyran in normal and excited state (A) and picture of the membrane (2 *μ*m, *c*_HEMA_ 25%) irradiated with UV-light through a mask (B).

In a post-polymerization modification step the membrane with the pHEMA layer was further modified with SP to introduce photo-responsive properties, as shown in scheme 1. In the post-polymerization modification the hydroxyl group of the pHEMA side chain was converted with an acid functionalized SP (SP-COOH). SP undergoes a reversible heterocyclic ring-opening causing a strong coloration and increase in hydrophilicity when irradiated with UV-light [39]. According to figure 4(A), the colorless closed form of SP turned to the colored and polar isomeric merocyanine form (MC), the initial closed form can be recovered by irradiation with white LED light. Therefore after the post-polymerization modification, photo-responsive membranes could be obtained. The initial colorless membrane changes its color to pink upon irradiation with UV. In figure 4(B) a photograph of the membrane irradiated through a mask is shown, clearly demonstrating a successful functionalization of the membrane with SP by showing a pattern.

### 3.2. Photo-responsive properties of the membranes

Next the photo-responsive properties of the membrane were analyzed in detail regarding the amount and stability of SP incorporated into the membranes.

#### 3.2.1. Amount of SP

The hydrolysis of SP derivatives back to the initial aldehydes used in the synthesis of these compounds is a known phenomenon during the photo-degradation process of SP [40]. The hydrolysis reaction of the SP moieties in the membranes to the aldehyde under basic conditions was used to determine the incorporated amount of SP in the membranes after the post-polymerization modification. Therefore, the SP containing membranes were dissolved in sodium hydroxide solution, after decomposition of the SP moieties; the concentration of the decomposition product 2-hydroxy-5-nitrobenzaldehyde in the solution was measured. The SP contents of the modified membranes are shown in table 2. It is already reported that the post-polymerization modification of pHEMA layer with SP only takes place on the surface of the pHEMA layer and therefore the SP molecules are only present on the membrane surface and not in the entire membrane [17]. Therefore, it turned out that the SP content seems to be independent on the thickness of the pHEMA layer or membrane morphology but is more determined by the steric hindrance of the bulky SP molecules already attached to the membrane surface preventing further attachment of additional SP molecules in their surroundings.

#### 3.2.2. Switchability of the membranes modified with SP

**Figure 5.**
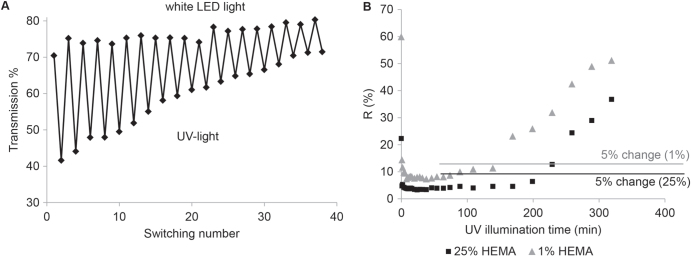
Reversibility of color switching (A) and fading rate under UV irradiation of the membranes functionalized with spiropyran (B).

The reversibility of the photo-responsive switching of the incorporated SP molecules from the colorless, non-polar SP state to the colored, polar MC form was analyzed. Figure 5(A) presents the reversible photo-responsive switching of the post-modified SP-containing membranes for 40 times by alternately illuminating the membrane for one minute with UV-light and for two minutes with white LED light. Photo degradation of SP in UV-light is a well-known problem for SP-based photo-responsive systems [41]. Indeed, after several switches the SP starts to partially decompose resulting in a decreased transmission. Nevertheless, the membrane was illuminated within this experiment with UV-light for altogether 20 min and still showed a reversible switching, which is already a comparable high value for a SP-based polymeric system [41].

#### 3.2.3. Stability

A slow ring-closing reaction of the MC form, equivalent to a comparable stable excited SP form, enables the use of pulsed UV-irradiation, reducing the irradiation time and thus also lowering the photo degradation of SP and prolonging the membranes lifespan. From the literature it is known that the chemical environment of the SP molecules has a major influence on the speed of the decoloration reaction in the dark, and that a more hydrophilic surrounding stabilizes the MC form [8, 42].

To explore the ring-closing kinetic of the membranes modified with SP the decoloration kinetics in the dark was measured. Therefore the membrane was illuminated once with UV-light and then UV/Vis spectra were recorded as a function of time as shown in figure S4. The initial decoloration rate slowed down after 25 min. Nevertheless after 25 min the difference in transmission between the colored MC form and the colorless SP form was still more than 70% of its original value, which demonstrates that the ring closing reaction is slow enough to allow a pulsed UV-irradiation. So the time period between two UV-light pulses can be chosen very long, protecting the membrane from photo degradation.

Also the stability of the SP dye under permanent UV-irradiation as a function of the polymer layer thickness was tested. In figure 5(B) the fading rate of the membrane modified with a HEMA concentration of 1% and 25% is shown. The first point in the graph corresponds to the membrane in the ground state before UV-irradiation, after approximately 15 min of irradiation the SP dye is fully excited. The excitement of the SP of the membrane modified with 1% HEMA concentration starts to decrease after 140 min whereas the excitement of the SP in the membrane with the thicker polymer layer (25% HEMA concentration) is stable for more than 200 min. In summary the fading rate of the membranes strongly depend on the amount of coating. The resistance to photo degradation of SP is strongly dependent on the chemical surrounding of the dye. More electron withdrawing groups are expected to stabilize the charged MC form to prevent photo degradation [40]. In the comparably thick pHEMA layer the SP moieties have predominantly pHEMA in the surrounding, whereas in the thin pHEMA layer also the polyester membranes are present influencing the stability of the SP upon UV-irradiation. Nevertheless these systems with a grafted pHEMA layer on polyester membranes and post-modified with SP showed an exceptional stability upon UV-irradiation compared to other systems based of the photo-responsive SP derivatives [13].

### 3.3. Permeability

The permeability of the membranes is their most important property in terms of an application as a transdermal delivery system. Caffeine was chosen in the permeability studies as model compound. It represents a group of small polar active components for possible applications of the photo-responsive membranes in drug delivery systems. The permeability of the membrane after illumination with UV-light is expected to increase according to the increased hydrophilicity of the charged MC form of SP [17, 18]. The increased hydrophilicity favors the interaction of caffeine with the membrane resulting in an increased flux of caffeine molecules through the pores of the membrane; whereas the more hydrophobic form of SP keeps the caffeine away from the pores due to repelling of the polar caffeine molecules. Changing the hydrophilicity of a membrane influences the wetting behavior of the pores. If the pore surface is hydrophilic, aqueous solutions pass through easily. If the pore surface is changed to a hydrophobic state, less aqueous solution is able to pass through [23].

The change of membrane permeability in dependence on the tunable wetting behavior of the pores possesses great advantages over systems where the change in membrane permeability is achieved by change in the geometric profile of the pores. Here the pores change their diameter in dependence on external stimuli, which can be a very challenging task, because the stimuli-responsive layer must on the one hand be thick and on the other hand carefully adjusted to the pore diameter to achieve a steric hindrance. In our system the change of hydrophilicity of the SP influences the surface tension of a membrane and therefore already a very thin coating is sufficient to achieve a change in permeability of the membrane. Moreover, due to the fact that the responsive polymer layer is located only on the membranes surface and not in the pores, no change in pore diameter or adjusting of the polymer layer thickness to the pore diameter is necessary in our approach.

**Figure 6.**
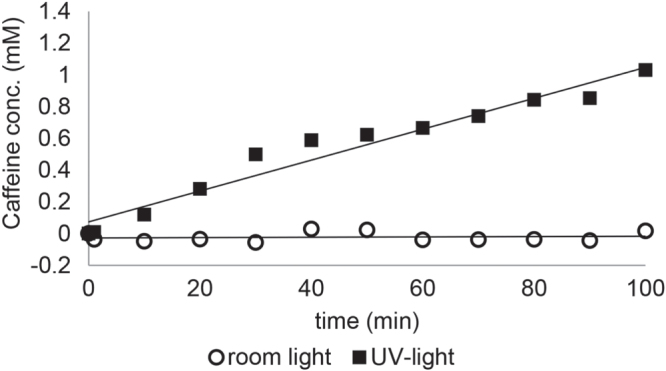
Mass transport rate of membrane (0.2 *μ*m, *c*_HEMA_ = 10%, polymerization time 60 min) under room light and UV-light.

The flux through the membranes measured under UV-light and room light are summarized in table 2. Exemplary, the flux of caffeine through a membrane with a pore diameter of 0.2 *μ*m and a HEMA concentration of 10% under UV-light and room light is shown in figure 6. This membrane showed an on–off behavior in terms of the flux of aqueous caffeine solution. Under room light even after 100 min no significant caffeine flux was detected, but under UV irradiation the membrane became permeable and a flux of caffeine through the membrane was detected. In comparison in figure S5 the flux of caffeine through a membrane with a pore diameter of 1 *μ*m (A) and 2 *μ*m (B) with a HEMA concentration of 25% under UV-light and room light is shown. Here the membranes exhibit a low–high permeability behavior. Under room light the membranes showed a clearly reduced caffeine flux compared to the flux under UV-irradiation. In general, the caffeine flux through the modified membranes could be tuned between 0 and 14 10^−9^ mol s^−1^ cm^−2^. The basic caffeine flux through the membranes in the ground state of SP was influenced by the pore diameter of the initial membrane, the applied HEMA concentration and the polymerization time. It turned out that the flux increases with very low HEMA concentrations (<1%) compared to the unmodified membranes due to an increased hydrophilicity of the pHEMA layer compared to the initial polyester membrane. But for higher HEMA concentrations (>1%) the flux decreases with increasing pHEMA layer, assigned to an increasing hindrance of the flux of caffeine through the pores caused by the polymer layer. In the case of membranes with a pore diameter of 0.2 *μ*m the pHEMA layer completely covers the membrane surface, here the decrease in permeability is caused by the diffusion of the small caffeine molecules through the pHEMA brushes to reach the pores of the membrane. Whereas for membranes with a pore feature size of 1 or 2 *μ*m the porous structure of the membrane was still present. In this case the pHEMA layer causes an increased blocking of the pores. Accordingly, the flux rises with increasing pore diameter of the applied membranes. However the complete covering of pores for membranes with a pore diameter of 0.2 *μ*m and the partial blocking of the pores for membranes with a pore diameter of 1 or 2 *μ*m show similar influences on the permeability behavior of the membranes, since membranes, which were prepared under the same conditions in terms of polymerization time and HEMA concentration and just differ in their pore diameter show all the same trend in the permeability behavior. It shows that the partial (or complete) covering of the pores has a smaller influence in the permeability behavior than the polarity of the membrane (it is more hydrophilic after UV irradiation).

A decrease in permeability of the membranes was also observed for an increasing polymerization time: the longer the polymerization time, the thicker the pHEMA layer and therefore a lower flux of caffeine through the membrane was observed.

After UV irradiation all SP-containing membranes showed a pronounced increase of permeability through aqueous caffeine solution with a maximum change in permeability of more than 1000%. For the membranes with a pore diameter of 0.2 *μ*m and an applied HEMA concentration of 10% we observed an on–off behavior. Here the membranes showed no detectable permeation of caffeine through the membrane under room light whereas after irradiation with UV-light the membranes became permeable. The other membranes exhibit a low flux of caffeine already under room light and after irradiation with UV-light the flux was remarkably increased.

Overall, the basic permeability of the membranes under room light could be tuned by the pore diameter, different HEMA concentrations and polymerization time; in addition these membranes showed an increase flux of aqueous caffeine solution, when irradiated with UV-light. Besides the good adjusting of the permeability properties of the membranes by the modification by ATRP of HEMA and post modification with SP, the big advantage of using modified track etched membranes lies in the high mechanical stability. For example, the stability compared to photo-responsive membranes consisting of amphiphilic conetworks, synthesized and published earlier from our group, was remarkably improved [11]. This allows a convenient handling without rupturing or destroying of the membranes, which is, besides the adjustable permeability, an important feature for possible applications in the field of drug delivery or sensing systems.

## 4. Conclusions

We developed a straightforward approach to produce photo-responsive membranes based on commercially available track etched membranes. Plasma-assisted functionalization followed by ATRP-initiator binding on the membrane surface with subsequent surface initiated ARGET–ATRP resulted in a controlled polymerization of a pHEMA layer on the membranes. The polymer layer thickness could be controlled by the applied HEMA concentration and the polymerization time. Further modification of the membranes with the photo-responsive SP molecules introduced photo-responsive properties into the membranes. We demonstrated that the basic permeability of the photo-responsive membranes in terms of flux of aqueous caffeine solution could be easily tuned by the pore diameter, applied HEMA concentration and polymerization time. Furthermore the tested membranes showed an increased flux after irradiation with UV-light (maximum > 1000%) with either an on–off behavior, or a low–high permeability behavior. The exceptional permeation switchability and stability of SP functionalized membranes enables possible applications in the field of drug delivery systems, sensors or filtration materials. The possibility of easy changing the properties of the membrane by changing the polymerization conditions will specially allow their use in tailor-made products for niche applications, where on-demand switching is needed to adapt for changing environmental requirements.
